# ezTrack: An open-source video analysis pipeline for the investigation of animal behavior

**DOI:** 10.1038/s41598-019-56408-9

**Published:** 2019-12-27

**Authors:** Zachary T. Pennington, Zhe Dong, Yu Feng, Lauren M. Vetere, Lucia Page-Harley, Tristan Shuman, Denise J. Cai

**Affiliations:** Department of Neuroscience, Icahn School of Medicine at Mount Sinai, New York, USA

**Keywords:** Software, Fear conditioning, Motor control

## Abstract

Tracking animal behavior by video is one of the most common tasks in the life sciences. Although commercial software exists for executing this task, they often present enormous cost to the researcher and can entail purchasing hardware that is expensive and lacks adaptability. Additionally, the underlying code is often proprietary. Alternatively, available open-source options frequently require model training and can be challenging for those inexperienced with programming. Here we present an open-source and platform independent set of behavior analysis pipelines using interactive Python that researchers with no prior programming experience can use. Two modules are described. One module can be used for the positional analysis of an individual animal, amenable to a wide range of behavioral tasks. A second module is described for the analysis of freezing behavior. For both modules, a range of interactive plots and visualizations are available to confirm that chosen parameters produce the anticipated results. Moreover, batch processing tools for the fast analysis of multiple videos is provided, and frame-by-frame output makes alignment with biological recording data simple. Lastly, options for cropping video frames to mitigate the influence of fiberoptic/electrophysiology cables, analyzing specified portions of time, and defining regions of interest, are readily implemented.

## Introduction

The ability to process videos of small animals and automatically score their behavior is crucial to a number of common tasks in the life sciences – be it measuring the locomotor activity of an animal, defining its position in an arena, quantifying its interactions with an object, or assessing its engagement in defensive behaviors like freezing. Still, despite the nearly ubiquitous need for automated video analysis of this sort, there are substantial barriers to accessing these functions. One is cost – existing commercial software can cost several thousand dollars. Another is flexibility – commercial software often constrains the experimenter to particular hardware, operating systems, and video file types. The last is usability – while often powerful, existing free software can sometimes require substantial programming experience to implement and can involve complex algorithms^1^.

To overcome these hurdles, we developed a simple, free, and open-source video analysis pipeline that (1) is accessible to those who have no programming background, (2) provides a wide array of interactive visualizations, (3) requires a minimal number of parameters to be set by the user, (4) produces tabular data in accessible file formats (e.g. csv), (5) accepts a large number of video file formats, and (6) is operating system and hardware independent. At the same time, being open-source, it allows users to modify the underlying code as they see fit.

Our behavior analysis pipeline, ezTrack, has two modules. The first is a module for analyzing an animal’s location throughout a session, the distance it travels, and the time it spends in user-defined regions of interest (ROI). The second allows the user to analyze freezing behavior, most relevant to the study of fear and defensive behavior. For both modules, options for outputting frame-by-frame data as well as time-binned summary reports are provided. Additionally, both modules allow the user to either process individual videos with extensive visualizations of the results to aid in parameter selection, or to process large numbers of files simultaneously in a batch. Lastly, users can easily crop the frame of their videos and define the range of frames to be processed in order to remove the influence of cables attached to the animal or other unwanted objects that might enter into the field of view. With this simple toolkit, a vast amount of automated behavioral analyses are readily performed.

## Results

ezTrack was designed to be implemented in iPython/Jupyter Notebook. Using Jupyter Notebook, the code is organized into “cells” – discrete, ordered sections that can be independently run by the user. Critically, instructions that inform the user what each cell does from a conceptual standpoint as they step through the code, as well as how to modify code when needed, precede each cell of code (Supplementary Videos 1, 2). That said, the core algorithms are implemented in separate Python scripts (.py files), so that inexperienced programmers only have to set the values of a few key variables/parameters (e.g. the folder in which files are stored or a threshold value), and choose whether they want to run a particular cell or not. This balances the user interface between usability and flexibility – the user can understand the algorithms conceptually without reading all of the code, while maintaining complete freedom to modify the algorithms if desired. Moreover, the output of running each cell is displayed directly below it so that the user can view the results of each cell of code that they run. To further leverage this, we provide numerous interactive plots and videos at critical steps to visually show the user the effect of algorithms/parameters, making the use of code intuitive. Tutorials of how to step through the code are presented in Supplementary Video 1 (Location Tracking Module) and Supplementary Video 2 (Freeze Analysis Module).

### Location tracking module

ezTrack’s Location Tracking Module assesses an animal’s location across the course of a single, continuous session. The Location Tracking Module can be used to calculate the amount of time an animal spends in user-defined ROIs, as well as the distance that it travels. It uses the animal’s center of mass to determine the animal’s location in each frame of the video (See Supplementary Video 1 for tutorial and Supplementary Video 3 for tracking example).

**Figure 1:**
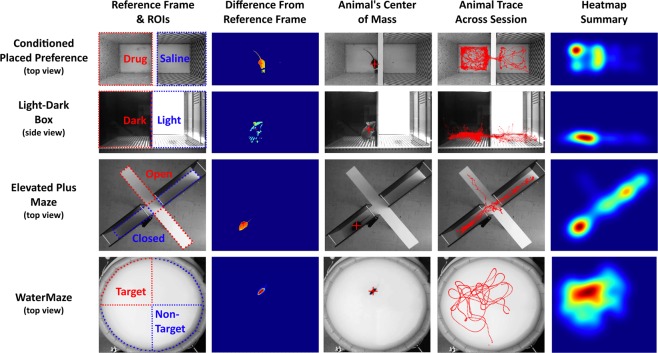
ezTrack Location Tracking Module. Using ezTrack’s Location Tracking Module, an animal’s center of mass is tracked across the session. After defining a reference frame and ROIs with a drawing tool (left-most panel), each frame is compared to the reference frame, taking their difference (2nd panel). From these differences, the animal’s center of mass is calculated (3rd panel, crosshair indicates center of mass). The animal’s center of mass is then saved, as well as displayed atop the reference frame for visual inspection of the results (4th panel). Interactive summary images, including heatmaps (5^th^ panel) are also output. All images come directly from ezTrack output.

To validate that ezTrack’s Location Tracking Module works for a wide range of behavioral assays, we analyzed videos of mice being tested for their preference of a cocaine-paired chamber (conditioned place preference), their preference for the darker side of a two-chamber box (light-dark test), their preference for the closed arms of an elevated plus maze, and their preference for the quadrant of a water maze that formerly contained a hidden platform (Morris water maze) (Fig. 1). As can be seen in Fig. 1, ezTrack was able to track the position of animals in all of these assays, despite different lighting conditions, arena sizes, and camera orientations. It is particularly noteworthy that ezTrack worked well with the light-dark box because using commercial software it is frequently difficult to maintain tracking of an animal when background lighting conditions change, or when there is generally low contrast between the foreground and background. Moreover, as demonstrated in Supplementary Video 3, ezTrack is quite robust to other objects that might enter the field of view. Provided the interfering object does not directly overlap with the animal in the field of view, tracking is maintained.

**Figure 2:**
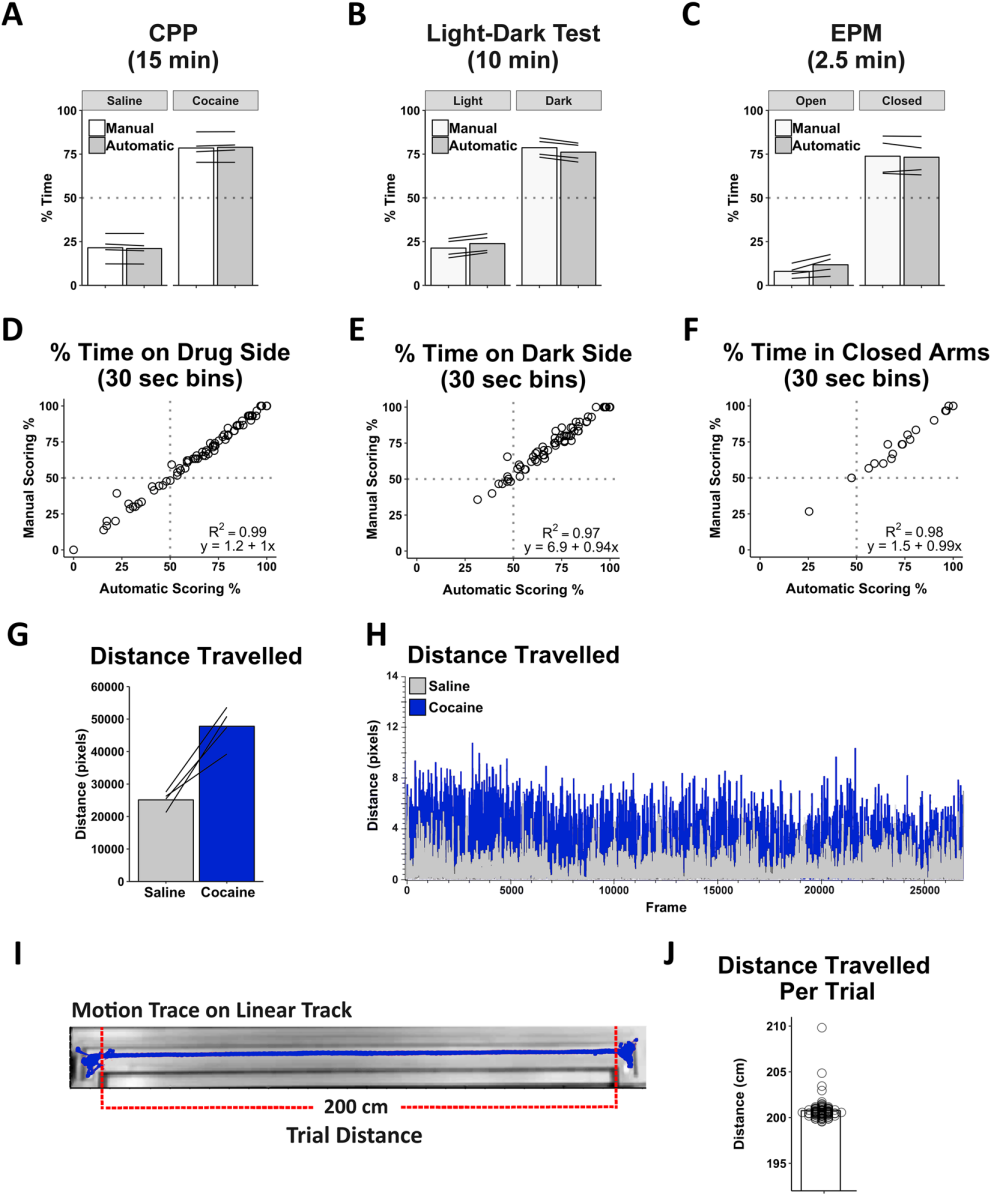
ezTrack Location Tracking Module Validation. Automated analysis of cocaine conditioned place preference (n = 4 animals), light-dark box (n = 4 animals), and elevated plus maze (n = 4 animals) rendered nearly identical results as scoring by hand, both when looking at mean session data (**A**–**C**), and when session data was broken down into smaller time segments (**D**–**F**), demonstrating ezTrack’s accuracy. Additionally, ezTrack reliably detects changes in locomotion, with increased motion in conditioned place preference following cocaine administration (**G**–**H**). Further validating ezTrack distance measurements, across 93 trials traversing a 200 cm linear track (**I**), ezTrack consistently indicated that an animal ran just over 200 cm (**J**).

To provide a quantitative validation of the Location Tracking Module, using ezTrack’s ROI drawing tool, we assessed the amount of time animals spent in ROIs for each task, and compared this with results of manual scoring. As can be seen in Fig. 2A–F, there was a high correlation between automated and manual scoring for cocaine place preference (R^2^ = 0.99, y = 1.2 + 1x), the light-dark test (R^2^ = 0.97, y = 6.9 + 0.94x) and the elevated plus maze (R^2^ = 0.98, y = 1.5 + 0.99x).

Another useful tool provided by ezTrack’s Location Tracking Module is its calculation of the distance an animal moves on a frame-by-frame basis, derived by taking the Euclidean distance of the animal’s center of mass from one frame to the next. We first examined conditioned place preference training data, in which animals were given either saline or cocaine. Using ezTrack, we were able to clearly track cocaine-induced hyperlocomotion (Fig. 2G–H. t_3_ = 6.4, p < 0.01). Although ezTrack calculates distance in pixel units by default, the user is also able to easily convert pixel distance measurements to other physical scales. Using point and click options, the user is able to specify any two points on the video frame and define the distance between them in the scale of their choice. ezTrack will then convert measurements that are in pixel units to the desired scale, while retaining pixel measurements as well. In order to calculate the accuracy of distance measurements, we quantified the distance of a well-trained animal running 93 trials on a 2-meter linear track (Fig. 2I–J). Because the track only allows for forwards motion, this allowed us to compare the distance ezTrack records to the actual distance travelled by the animal. Across trials, the distance travelled by the animal was nearly identical to the expected value of 200 cm (mean = 200.75 cm, min/max = 199.5/209.8; Fig. 2J). Because the minimum distance an animal can travel is 200 cm, this tight and slightly positively skewed distribution is exactly what one would expect from ideal tracking.

Beyond many researchers’ interest in locomotion as an experimental variable, the frame-by-frame trace of distance that is automatically output by ezTrack is also useful in detecting anomalies in tracking (Supplementary Fig. 1). If something enters into the field of view and biases the center of mass, a large deflection in the distance traveled is anticipated. In this way, ezTrack alerts users to potential failures of tracking, a feature often not provided by other software. An example of this can be seen in Supplementary Fig. 1, in which an experimenter’s hand repeatedly entered into the camera’s field of view to pipette a liquid reward for the animal. If tracking is biased by objects that enter into the field of view, the experimenter is able to implement a location weighting algorithm that reduces the influence of outside objects and can in many cases restore tracking (Fig. S1 and Supplementary Video 3; see Methods for details).

**Figure 3:**
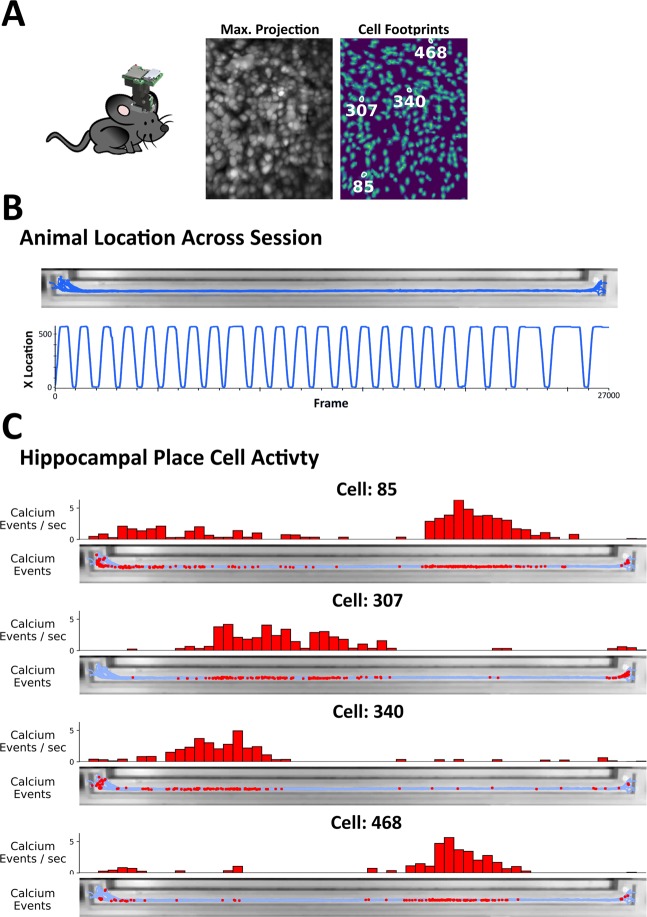
Alignment of Neurophysiological Data with ezTrack Results. A single mouse ran back and forth on a linear track wearing a Miniscope in order to track hippocampal place cells. (**A**) Animal with head-mounted Miniscope. Image on left shows max projection of Miniscope recording (for each pixel, max. value across session). Image on right shows isolated cell locations. Exemplary ‘place cells’ are highlighted. (**B**) Using ezTrack, animal’s spatial position was examined during a single session. Top image shows animal position superimposed on linear track. Bottom image shows animal’s x position across a session. Note smooth tracking throughout. (**C**) After deconvolving calcium activity, calcium events were aligned with spatial position to identify putative place cells. Top histogram shows normalized cell activity across track. Bottom image shows imposition of calcium events atop linear track.

By default, ezTrack’s Location Tracking Module outputs frame-by-frame data in convenient csv files, making alignment with neurophysiological recordings a simple task. As a demonstration of this, we aligned single-photon *in vivo* calcium imaging with a Miniscope recording of hippocampal sub-region CA1 with location tracking results obtained from video of a mouse running back and forth on a 2-meter linear track. As can be seen in Fig. 3 and Supplementary Video 4, we were able to define the location of each calcium event along the location of the linear track, and from this discern each cell’s place field^2^.

### Freeze analysis module

**Figure 4:**
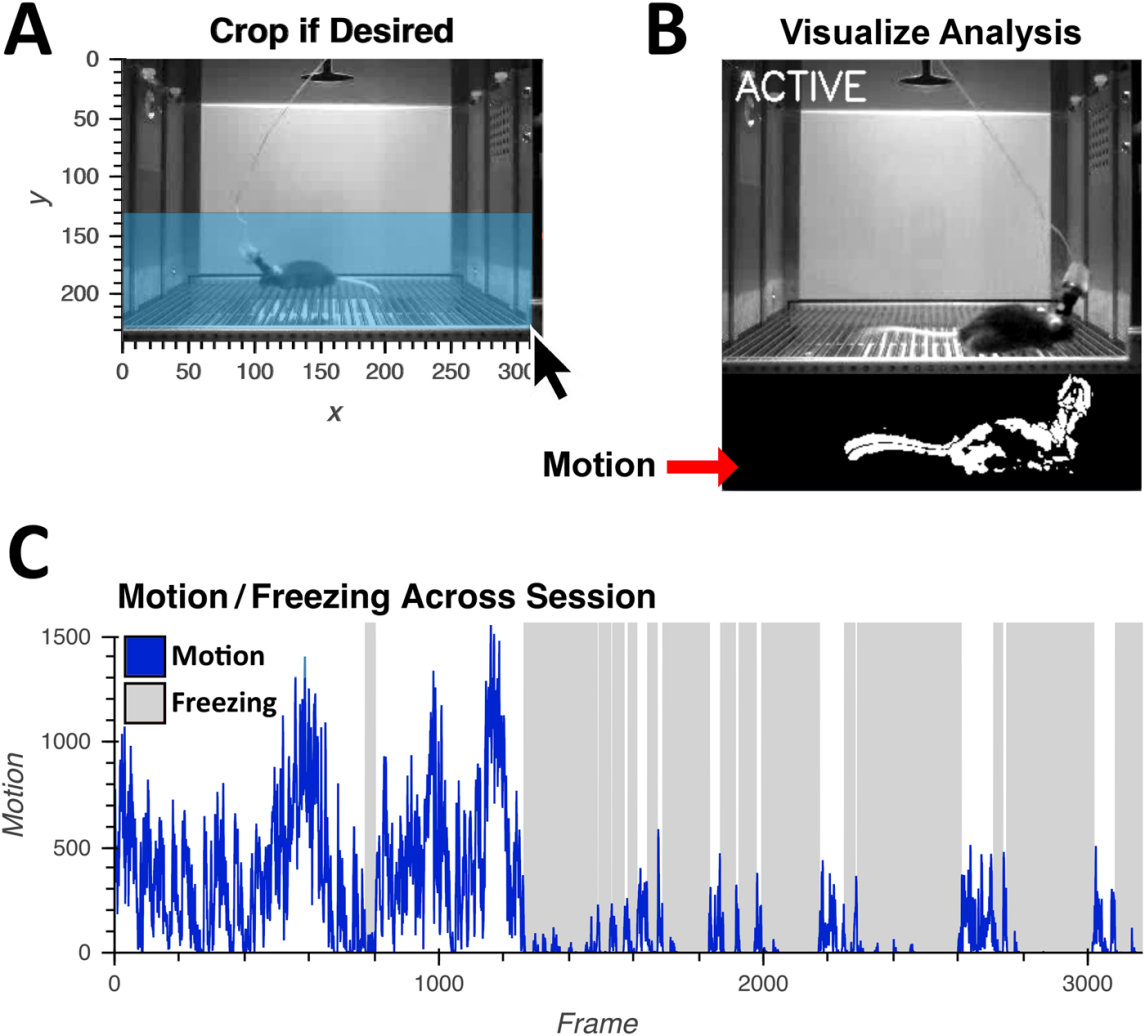
ezTrack Freeze Analysis Module. Using ezTrack’s Freeze Analysis Module, an animal’s motion and freezing are processed. (**A**) Using point and click options, ezTrack’s Freeze Analysis Module allows the user to crop the the frame to remove the influence of cables. (**B**) After analyzing data, segments can be played back to visualize scoring of motion and freezing (See Supplementary Video 5). Parameters can then be adjusted to conform to the experimenter’s judgment. (**C**) Motion (blue) and freezing (gray) is plotted by ezTrack and frame-by-frame and binned summary data can then be saved to csv files.

ezTrack’s Freeze Analysis Module allows the user to measure freezing, employing its most common definition: the absence of movement, barring respiration. Similar to commercial automated software like VideoFreeze^3^ and FreezeFrame, ezTrack first measures the motion of the animal by assessing the number of pixels whose grayscale change exceeds a particular threshold from one frame to the next. Subsequently, an animal is scored as freezing when motion is below a user-defined threshold for a user-specified amount of time. The Freeze Analysis Module provides extensive visualizations in order to set thresholds which conform to manual inspection of videos. This includes interactive graphs in which the user can view the interrelationship of motion and freezing (Fig. 4), as well as videos that allow the user to see what is being picked up as motion/freezing (Supplementary Video 5). Additionally, provided side-view recording is performed, ezTrack’s point and click cropping tool allows one to remove the influence of any fiberoptic/electrophysiology cables attached to the animal (Fig. 4), which can continue to move even when the animal is freezing and bias measures of freezing. A tutorial of how to use the Freeze Analysis Module is provided in Supplementary Video 2.

**Figure 5:**
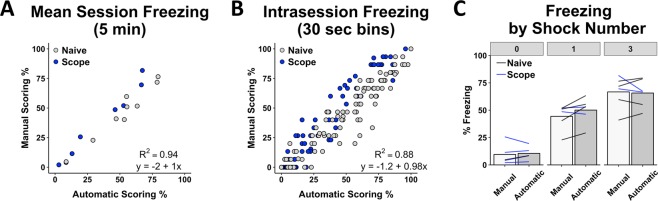
ezTrack Freeze Analysis Module Validation. The results of ezTrack’s Freeze Analysis Module were compared to those obtained from manual scoring of freezing. Automated analysis of freezing behavior was highly correlated with manual freezing, both when examining (**A**) average freezing across a 5 min session (n = 17 animals), (**B**) and using 30 second time bins (n = 255 samples). (**C**) Moreover, both manual and automated scoring show increased freezing with the number of shocks animals previously received, and relative between-mouse freezing levels are preserved (each mouse is represented by a line). Naive animals had no surgical experience whereas Scope animals had a Miniscope attached during recording.

To validate the accuracy of the Freeze Analysis Module, we analyzed videos from animals that underwent associative fear conditioning. Animals had undergone a varying number of conditioning trials (0, 1 or 3 footshocks; each 1 mA, 2 sec in duration) and were then placed back into the conditioning chamber the subsequent day to assess their freezing as an indicator of fear. We compared the percentage of time spent freezing for the final test session, determined either by manual scoring or using ezTrack. Ratings of average freezing for the entire 5-minute session were tightly correlated (Fig. 5A; R^2^ = 0.94, y = −2 + 1x), as were ratings when we divided the test session into 30 second bins (Fig. 5B; R^2^ = 0.88, y = −1.2 + 0.98x). Moreover, using both methods, group-level freezing paralleled the number of shocks received (Effect of shock: F_2,14_ = 35.6, p < 0.001), and automatic scoring did not bias freezing across groups with varying average freezing levels (Effect of scoring method: F_1,14_ = 1.42, p = 0.25; Scoring method x shocks: F_2,14_ = 1.71, p = 0.22). Lastly, ordinal differences in freezing between animals was well-preserved (Fig. 5; Spearman’s R = 0.95). Of note, half of these animals were wearing head mounted Miniscopes^4^ and had a cable extending from the top of the scope during recording. Many researchers have resorted to hand-scoring similar videos because commercial software is unable to remove the influence of cables. By taking the simple solution of cropping the top of the video, ezTrack allows the user to circumvent this problem.

## Discussion

Above, we describe ezTrack, a flexible toolkit for the analysis of behavior, and demonstrate that ezTrack produces highly accurate results across an array of behavioral tasks and under varied experimental conditions.

The tasks presented should be thought of as a representative sample of the vast number of behaviors that could be analyzed with ezTrack. The Location Tracking Module could be used for nearly any task in which an animal is moving throughout a stable background (e.g. place preference, water maze, radial arm maze, infinity maze, open field, etc). Moreover, although the Freeze Analysis Module was designed for the study of defensive behavior, it could also potentially be used to analyze immobility in the forced swim task, or diurnal activity. Additionally, although ezTrack was tested here with mice, it could be used with any experimental species where the goal is to track the position of the animal.

ezTrack represents a substantial deviation from prior solutions to the problem of automated video analysis. The predominant solution – commercial software – has been relatively easy to use but costly and inflexible. Researchers are frequently bound to use particular hardware which may not always be compatible with their research questions. Moreover, the underlying code and its operations are often proprietary, prohibiting alterations by the researcher. Alternatively, free solutions like DeepLabCut^1^ are great because they have the ability to track a wide range of sophisticated behaviors. However, they have been difficult to implement for scientists with little programming/computational experience. Moreover, although deep learning algorithms allow for the analysis of more complex data sets, they also require prior model training based upon training sets. The generation of training sets that are representative and generalizable can be time-consuming and prone to error. ezTrack takes the middle road, providing a set of free tools that can be quickly implemented by anyone, allowing fast, automatic scoring of a seemingly endless number of data sets.

Both commercially available software (e.g. Ethovision, SMART, Video Freeze, FreezeFrame), and open-source options for behavior analysis (e.g. Bonsai) confine researchers to using a particular operating system, and often to particular video file formats. A major advantage of ezTrack is that it can be readily used on OSX, Windows, and Linux operating systems. Additionally, because ezTrack does not load entire videos into memory, memory and processing requirements are relatively small, making simple computers sufficient for use with ezTrack. In conjunction with ezTrack’s acceptance of a wide variety of video file formats, these factors make ezTrack’s integration into nearly any lab environment possible.

ezTrack will likely be particularly useful for researchers who combine behavioral monitoring with *in vivo* electrophysiological, optical and other biophysiological monitoring/manipulation techniques. First, commercial software does not readily output frame-by-frame data (e.g. Video Freeze), making it difficult to align biological and behavioral measurements at the necessary resolution. By default, ezTrack outputs this data in convenient csv files. Moreover, commercial software is frequently biased by cables that are attached to the animal. ezTrack permits the user to crop the frame of the video to remove the influence of these objects. Simple as they are, these features of ezTrack will make the research process much easier.

ezTrack is simple not only in its technical implementation, but in its conceptual nature (see Methods). Both the Location Tracking Module and the Freeze Analysis Module have very few parameters that must be set by the user and there are no hidden parameters in the underlying code. In combination with the rich set of visual outputs that ezTrack provides, it is easy for the researcher to understand the relationship between the data input and the data output, and thus, how parameters can be changed to improve tracking accuracy. The intuitive nature of ezTrack will provide transparency in its results in comparison to programs where data is processed within a ‘black box’. Furthermore, ezTrack’s simplicity facilitates modifications to the code for those who wish to do so. Along these same lines, ezTrack could be an incredible instructional tool for teaching students at many levels how to process video content.

Data reproducibility is another benefit of ezTrack. ezTrack’s use of Jupyter Notebooks, an html-based platform, makes it so that all selected parameters, the steps that were taken to process a dataset, and the output, can be saved in a highly readable pdf or html file (being a printout of the notebook the data was processed in). This stands in contrast to graphical-user-interface-based systems in which point and click options are not necessarily saved, and when they are, are not convenient to access. This makes it easier for the experimenter and others to go back and confirm how the data was processed.

ezTrack is not without limitations. In its current form, ezTrack measures the activity of a single animal and works predominantly by tracking the animal’s center of mass. This hinders the analysis of social interaction, of more specific sorts of interactions with objects (e.g. sniffing, grabbing, burying), and of fine motor movements. It is likely that the measurement of some of these behaviors – particularly fine motor movements – are better accomplished with more sophisticated algorithms^1^. However, other features, like tracking an animal’s direction and tracking multiple animals, can be added to ezTrack in the future as needed. Indeed, a major benefit of ezTrack being open-source is that it is relatively fast to update and distribute new features and modifications. For example, due to the simplicity of the computation, ezTrack could readily be adapted to work in real-time in conjunction with output devices. Although we have not included this option in this version, these and additional modifications could be incorporated in future versions of this behavioral analysis pipeline. In closing, ezTrack can fulfill a broad set of researchers’ needs and the open-source platform facilitates the flexibility of adding new functions in ezTrack. We welcome users to try it. It’s ez.

## Methods

### Installation and dependencies

Detailed instructions on ezTrack installation can be found on Github: github.com/DeniseCaiLab/ezTrack. In brief, after following the instructions for downloading Conda/Miniconda (a free package management system) the commands are given for installing both Jupyter Notebook and other free packages required for the code to be run. Subsequently, Python/iPython files can be downloaded from our Github account and run within Jupyter Notebook. Supplementary Videos 1 and 2 provide a brief tutorial for using Jupyter Notebook. The following opensource packages are used for video import, data manipulation, and interactive plotting: iPython^5^, OpenCV^6^, Pandas^7^, HoloViews^8^, SciPy^9^, Matplotlib^10^, Bokeh^11^, and NumPy^12^. Of note, package versions are maintained by Conda and controlled for in the install instructions. Package compatibility issues should therefore not arise. Nevertheless, we actively maintain our Github page and will provide updates to ezTrack as new features are added and new package versions become available. Additionally, we welcome feedback and bug reports on the ezTrack Github page, and encourage users to watch the ezTrack Github page in order to be aware of any new releases.

### Video acquisition suggestions

Although ezTrack works on a wide variety of videos, certain conditions will render tracking unreliable. Acquired videos must be captured with a mounted camera so that the field of view is fixed throughout the recording session. Likewise, although both tracking modules are compatible with numerous angles of recording (e.g. above the animal or to the side), it is critical that the directional relationship between the camera and the arena, as well as the distance between the camera and the arena, be maintained across the course of an individual experiment. Moreover, ezTrack works best under conditions where there is good contrast between the animal and the background – if it is hard for the human experimenter to see the animal, it will likely be problematic for ezTrack to calculate the center of mass of the animal and track its location. ezTrack was programmed to be robust against the influence of objects other than the animal in the field of view. Cropping of the video frame is supported, and the Location Tracking Module has a function that reduces the influence of objects that might enter the field of view: weighting the region surrounding where the animal was on the prior frame (described in detail below). Nevertheless, ezTrack will work best when only the animal and a stable environment are in the field of view. Moreover, the animal should not be able to exit and re-enter the field of view. With this in mind, one should not crop so stringently that the animal would disappear from the field of view if it rears – we find that much of the artifact produced by cables attached to an animal emerges some distance above the animal’s head, where slack in the line is greatest. If one is doing optogenetic experiments and light from the fiberoptic is likely to leak, we recommend using an infrared camera that will not pick up the wavelength of the stimulation light-source. In its current state ezTrack only tracks a single animal. Simultaneously recording multiple arenas with one camera is possible. However, for now, the experimenter will have to individually analyze each arena by cropping the other arenas out of the field of view. All batch processing features assume that the position of the camera, the position of the environment within the field of view, and the lighting conditions, are consistent across sessions. We have tested ezTrack with wmv, avi (mp4 codec), and mpg1 videos, but many more video filetypes are possible. If your particular filetype is not supported, there are several free video converters available online. Lastly, note that high definition videos can take longer to process and down sampling/compression of video may be desired.

### Location tracking module

#### General description

The Location Tracking Module tracks the center of mass of an animal within the field of view on a frame-by-frame basis. From this, the distance the animal travels and its time spent in particular ROIs are calculated. Video can be taken either looking down on the animal, or from the side, though side view recording can put distance measurements on an arbitrary scale. Using a drawing toolbox, the user is able to crop the field of view, and the user can also restrict the analysis of the video to a particular range (e.g. frames 500–1000). Additionally, the user is able to draw an unlimited number of ROIs, and for each frame ezTrack will determine if the animal is in a particular region or not. Notably, ROIs are allowed to overlap, allowing sub-regions to be analyzed. While running the code, interactive plots are generated which allow the user to view both the distance travelled across the session and traces of where the animal went. As an added feature, if video files are small (e.g. 100 × 100 pixels), or if they are odd dimensions (e.g. 30 × 1000 pixels), ezTrack allows the user to stretch the video horizontally/vertically for presentation purposes. The frame by frame location of the animal in x/y coordinates, as well as whether the animal is in each defined ROI, are saved to a csv file. If the user wants summary information, options are provided for specifying time bins, and a summary file will be generated giving the distance travelled in each time bin, as well as the proportion of time spent in each defined ROI. Once the user is confident that tracking is working well for individual videos, they can then perform batch processing, in which every video file in a folder will be processed.

#### Methods for location tracking

Location tracking is accomplished by comparing each frame in a video to a reference frame where the animal is not present in the environment. ezTrack supports two ways to define this reference frame. The easier, faster and often more reliable option is to generate a reference frame from the video being analyzed (i.e. the video with the animal). ezTrack will generate a reference frame based upon a random sample of frames across the video (default = 100). For each pixel in the field of view, the median value across the selection of frames is taken. Critically, this median image will likely not contain the animal unless the animal is in one location for over 50% of the session. If this occurs, the user can alternatively supply a separate video of the same environment with the same lighting conditions and with the environment in the same position in the field of view, but without the animal. When batch processing, the first option is always used, such that the reference frame for each video is generated from that video.

Next, to determine the center of mass for the animal, for each frame the grayscale intensity difference from the reference frame is calculated on a pixel-by-pixel basis. This can be done by taking the absolute difference between the two frames, which permits tracking regardless of whether the animal is lighter or darker than the background. Alternatively, one can impose the assumption that the animal is lighter or darker than the background (subtracting the reference frame from each frame or subtracting each frame from the reference frame, respectively). The latter approaches can be more reliable under certain circumstances. In order to mitigate the influence of random, low intensity fluctuations in pixel intensity values when calculating the center of mass, which we find can greatly bias accuracy, pixel differences are then thresholded so that only the difference values above a user-defined percentile of all pixel differences are considered. We have used the 99^th^ percentile and this has worked incredibly well. Notably, because this criterion scales relatively, the animal will generally be tracked equally well if it moves from a high to low contrast area of the arena, as in the light-dark box (see Fig. 1). Lastly, the center of mass on these values is calculated, returning the x/y coordinates of the animal.

In the event that something enters the field of view, biasing the center of mass to be between the animal and the interfering object, ezTrack allows the user to weight the pixels surrounding the animal’s previous location to mitigate this bias. For each frame, a weight (ω) can be applied to a square window surrounding the center of mass of the animal on the previous frame, the size of which is set by the user. Pixel difference values outside of the window are multiplied by (1 − ω), where ω is set to between 0 and 1. Thus, if ω = 1, pixel value differences that are outside the window will be set to 0. By contrast, if ω = 0, the interior/exterior of the window carry equal weight. In this way, the influence of pixel changes outside the window can be restrained to a minimal level controlled by a single parameter, ω. To avoid the potential issue that the animal moves out of the window (such as when the recording starts before the animal is placed in the field of view), ω can also be set to something between 0–1, which will allow the window to ‘snap’ back to the animal. We have used ω = 0.8–0.9 with good results. See Supplementary Fig. S1 for an example.

Lastly, the distance the animal traveled from one frame to the next is calculated as the Euclidean distance between the center of mass on adjacent frames.

Notably, the only parameter required to be set by the user is the percentile for setting pixel difference values to zero. The other two parameters – setting a weight and a window size for weighting difference values in the vicinity of the animal’s location on the prior frame – are optional.

### Freeze analysis module

#### General description

In order to detect freezing, ezTrack first measures the motion of an animal by calculating the number of pixels whose grayscale value changes from one frame to the next. However, because most videos display many small fluctuations in pixel values, even with a static scene, a cutoff must first be set to separate pixel changes that are attributable to the animal moving versus those that would occur with no animal in the box. ezTrack provides a calibration tool for examining the distribution of grayscale changes for a video of an empty box and provides a suggested cutoff. In light of this, it is imperative that the user generate short videos of the conditioning chamber without an animal in it during the course of their experiment so that basal pixel fluctuation can be equated between experimental sessions and the calibration video. Following calibration, the user can analyze videos of animals in that same environment. After measuring the motion of an animal, freezing can be calculated by assessing the amount of time the animal’s motion drops below a user-defined threshold. See Supplementary Video 2 for a tutorial.

#### Methods for freeze analysis

Calibration: To alleviate small fluctuations in pixel values attributable to noise, we found it helpful to first implement a gaussian filter (sigma = 1) on each image. Then, we calculate pixel-wise differences on consecutive frames, and count the number of pixels whose difference value exceed a certain cutoff (the motion threshold, or MT). To define MT, the user provides a short video of the recording environment without an animal (~10 sec). ezTrack will then calculate the distribution of pixel grayscale changes across this time period. It is then possible to set MT based on this null distribution. With our particular setup, we found that twice the 99.99^th^ percentile worked well. However, ezTrack provides a histogram of the distribution of difference values and also provides the user with the ability to see what is being picked up as motion side by side with the original video for a given MT (Supplementary Video 5). If the user senses too much noise or not enough signal is being detected, they can modify MT as they see fit. The only recommendation is that they maintain this threshold across all animals in an individual experiment. Provided video settings are not changed across days, we find that the MT for a given environment is very stable.

Measuring Motion/Freezing: Once the user defines MT, they can then analyze motion and freezing on an individual session. Each frame is gaussian filtered and the number of pixels whose grayscale change relative to the prior frame exceeds MT is determined. The user can then set a threshold for the number of changed pixels below which freezing is declared – the freezing threshold, or FT. As an additional criterion, a minimum freeze duration can be set (e.g. half a second). Thus, for any given succession of frames in which the number of changed pixels falls below FT for a period of at least the minimum freeze duration, the animal will be marked as freezing. FT can be set by the user by first inspecting plots of motion (i.e. changed pixels) across the session and noting the values corresponding to markedly low points, presumably freezing. After selecting a tentative FT, they can then watch video in which they can inspect the accuracy of their threshold and adjust it as they see fit (Supplementary Videos 2 and 4). When the user is satisfied with a set of parameters, they can then proceed to process several videos in batch. When batch processing, individual csv files containing frame-by-frame motion and freezing values are saved for each video. Additionally, the user can define time bins and a summary file containing the average freezing and motion during each time bin, for each video, will be returned.

### Behavioral testing and manual scoring

#### Manual scoring

All manual scoring was performed using a time-sampling procedure in which the instantaneous location/freezing of an animal was assessed every 1–2 sec (2 sec for freezing, 1 sec for all other behaviors). Freezing was defined as the absence of movement, barring respiration and minor bobbing of the head. Notably, because this is a time-sampling procedure, an animal only needed to be freezing at the time of sampling, and not during the entire 2 seconds preceding the sample. This method is in line with classic analysis of freezing behavior^3,13–15^. To define an animal’s location in the chamber, the location of the center of the animal was judged. Categorical responses were then turned into proportions by examining several responses over time.

#### Animals

Adult male C57/BL6J mice from Jackson Laboratories were used for all testing. Animals were housed in a temperature, humidity and light controlled vivarium down the hall from the experimental testing rooms with lights on at 7 a.m. and off at 7 p.m. Food and water were available *ad libitum*, with the exception of the animal running on the linear track, for which water was restricted to maintain a body weight of 85–90%. Water deprivation consisted of allotting the animal ~1 mL of water per day, including water obtained during testing. Water not obtained during testing was given after the testing period. Animals were acclimated to handling for 5–7 days prior to training/testing for 1–5 min/day. All experiments were performed in accordance with relevant guidelines and regulations approved by the Institutional Animal Care and Use Committee of Icahn School of Medicine at Mount Sinai.

#### Behavior

Fear Conditioning. Animals were placed in a novel conditioning chamber (Med Associates VFC-008; 30.5 × 24.1 × 21 cm) with a distinct scent (5% Simple Green) and received either 0, 1, or 3 scrambled footshocks (1 mA, 2 sec). The first shock was administered after a 2 minute baseline and each subsequent shock was administered after 1 minute. Animals were removed 2 minutes following the final shock and returned to the vivarium. Unshocked animals were placed into the chamber for the same period of time as animals that received 1 shock. On a subsequent day, animals were placed back into this environment for a 5 minute test session in which no shocks were given. Behavior was recorded using an infrared camera (Med Associates) recording at 30 fps. Automated scoring with ezTrack used a 0.5 second minimum freeze duration and the tops of video frames were cropped to remove the influence of Miniscope cables, where applicable. The amount of time spent freezing was assessed. Animals wearing Miniscopes required a slightly different threshold than animals not wearing Miniscopes, but otherwise the same freezing threshold was used for all animals, which was determined by visual inspection of a subset of the videos. A total of 17 animals was used (No shock = 5, 1 shock = 6, 3 shocks = 6). 8 of these animals had Miniscopes implanted.

Conditioned Place Preference. Conditioning took place across two days. On each day, animals were confined to one side of a conditioning chamber (dimensions of each side: 20 × 20 × 29 cm) for 15 minutes immediately after receiving an injection of saline or cocaine (15 mg/kg, i.p.). They were placed on alternate sides across training sessions so that one side would be associated with cocaine. The following day, animals were placed back in the chamber for 15 minutes and allowed to freely explore its two sides. The amount of time spent in the cocaine-paired side was examined. Additionally, distance travelled during training was measured using ezTrack’s distance output, calculated as the Euclidean distance of an animal’s location on successive frames. A total of 4 animals was used.

Light-Dark Test. Animals were placed in a chamber (30.5 × 24.1 × 21 cm) with a small opening connecting its two sides, one side being brightly lit (~400 lumens), the other being dimly lit (~10 lumens). The front of the dark side, through which the animal was recorded, was covered in a red transparent film to prevent the spread of white light into the box; the front of the brightly lit side was translucent. In order to facilitate visibility of the animal, an infrared light and camera were used (hence, lighting in Fig. 1 does not reflect perceived lighting by the animal). The proportion of time spent on the dark side was measured. A total of 4 animals was used.

Elevated Plus Maze. Animals were placed at the center of an elevated, plus-shaped, platform with two walled arms (closed) and two unwalled arms (open) for two and half minutes. Each arm measured 29.2 × 7.6 cm, and the EPM was suspended 53.3 cm above the floor. The proportion of time animals spent in the closed arms was assessed. A total of 4 animals was used.

Water Maze. A circular tub (120 cm in diameter) filled with a combination of water and white paint was used. A platform (10 cm in diameter) was submerged in one quadrant and start locations were randomized to one of four equidistant locations. An animal was trained three times per day for six days. In each trial, the mouse was given 60 seconds to find the platform. If the mouse found the platform earlier than 60 seconds, the trial ended. If the mouse failed to find the platform, the trial terminated at 60 seconds. After each trial, the mouse was put on the platform for 15 seconds.

Linear Track. For linear track recording sessions, a water-restricted animal ran back and forth on a 2-meter-long linear track during a 15 minute session in order to earn water rewards (10 ul) that were alternately given at each end. The data presented come from a single session for a well-trained mouse. For validation of distance measurements, a trial was defined as beginning when the animal left one goal box (an outcropping at the end of each side of the linear track) and ended when it entered into the goal box at the opposite end of the linear track. Only trials in which the animal ran the full length of the linear track were included in the analysis of distance travelled.

### Miniscope imaging

A Miniscope was used to image calcium events in the CA1 region of the hippocampus as previously described^4,16^. The analysis of calcium recordings was performed using an in-house script based on existing algorithms^17,18^. The script used in this paper is available online: https://github.com/DeniseCaiLab/minian. The pipeline is described briefly here: First, the raw calcium imaging videos are passed through a median filter and subsequently an anisotropic filter frame-wise to reduce granular noise. A general background is estimated by convolving each frame with a mean kernel whose size is a few times larger than the expected size of cells, and this background is then subtracted from each frame to remove the vignetting effect. Next, a standard fft-based cross-correlation method is used to estimate and correct for translational motion in the video, followed by a demon-based algorithm to correct for residual non-rigid motion. Next, an over-complete set of “seed” pixels is generated by finding the local-maxima on the maximum projection of a randomly selected subset of frames. These seeds are further refined and merged based on properties of their corresponding temporal dynamics (e.g. peak-to-peak values or peak-to-noise ratios), after which these seeds are used to identify the initial regions of interest that may correspond to cells^18^. Lastly, the seeds-based initial regions of interest and their corresponding activities are fed into the CNMF algorithm as initial spatial and temporal terms, respectively^17^. The CNMF algorithm decomposes the video into a matrix of spatial footprints and a matrix of corresponding temporal activities for each cell. In addition, the CNMF algorithm models the temporal activities of cells as an auto-regressive process, and de-convolves the signals to give an estimation of underlying spiking activity. Due to the slow evolution of calcium signals, we use the de-convolved signals for further analysis of place cells. Due to imperfect regularization of spike counts during the estimation of the deconvolved signal, there are many low-amplitude, false-positive spikes. Thus, we performed a thresholding on the signals to get a binary variable representing whether a calcium event was observed on given frames. This threshold was chosen arbitrarily by observing the distribution of the deconvolved signals across cells. To find cells that are most likely coding for the location of the animal, we align the behavioral tracking results to the calcium recordings frame-wise using a nearest-neighbor approach. Then, we calculate spatial information content for each cell by treating the thresholded, deconvolved signals as “spike trains”^19^. Example cells with high spatial information content are shown in Fig. 3.

## Supplementary information

Supplementary Video Figure Captions

Supplementary Video 1

Supplementary Video 2

Supplementary Video 3

Supplementary Video 4

Supplementary Video 5

## Data Availability

Source code for ezTrack is available at github.com/DeniseCaiLab/ezTrack. All data presented in the manuscript is available upon request.

## References

[1] Mathis A (2018). DeepLabCut: markerless pose estimation of user-defined body parts with deep learning. Nat Neurosci.

[2] O’Keefe J (1976). Place units in the hippocampus of the freely moving rat. Exp Neurol.

[3] Anagnostaras, S. G. *et al*. Automated assessment of pavlovian conditioned freezing and shock reactivity in mice using the video freeze system. *Front Behav Neurosci* **4**, 10.3389/fnbeh.2010.00158 (2010). 10.3389/fnbeh.2010.00158 PMC2955491 20953248

[4] Cai DJ (2016). A shared neural ensemble links distinct contextual memories encoded close in time. Nature.

[5] Perez F, Granger BE (2007). A System for Interactive Scientific Computing. Computing in Science & Engineering.

[6] Bradski, G. The OpenCV Library. *Dr. Dobb’s Journal of Software Tools* (2000).

[7] McKinney, W. In *Proceedings of the 9th Python in Science Conference*. 51–56 (2010).

[8] PyViz. *HoloViews* (2018).

[9] Jones, E., Oliphant, T., Peterson, P. & others. *SciPy: open source scientific tools for Python*, http://www.scipy.org/ (2001).

[10] Hunter JD (2007). Matplotlib: A 2D graphics environment. Computing in Science & Engineering.

[11] Bokeh, D. T. *Bokeh: Python library for interactive visualization* (2018).

[12] Oliphant, T. E. *A guide to NumPy*. (Trelgol Publishing, 2006).

[13] Fanselow MS (1980). Conditioned and unconditional components of post-shock freezing. Pavlov J Biol Sci.

[14] Fanselow MS (1986). Associative vs. topographical accounts of the immediate shock freezing deficit in the rat: Implications for the response selection rules governing species-specific defense reactions. Learning and Motivation.

[15] Fanselow, M. S. & Lester, L. S. In *Evolution and Learning* (eds. Robert C. Bolles & M. C. Beecher) 185–211 (Erlbaum, 1988).

[16] Shuman, T. *et al*. Breakdown of spatial coding and neural synchronization in epilepsy. *BioRxiv* (2018).

[17] Pnevmatikakis EA (2016). Simultaneous Denoising, Deconvolution, and Demixing of Calcium Imaging Data. Neuron.

[18] Lu J (2018). MIN1PIPE: A Miniscope 1-Photon-Based Calcium Imaging Signal Extraction Pipeline. Cell Rep.

[19] Skaggs, W. E., McNaughton, B. L., Gothard, K. M. & Markus, E. M. In *Advances in Neural Information Processing Systems* **5**. 1030–1037 (Morgan Kaufman Publisher’s Inc., 1993).

