# Supplementary material for: ezTrack: An open-source video analysis pipeline for the investigation of animal behavior
Source: Sci Rep. 2019 Dec 27;9:19979. doi: 10.1038/s41598-019-56408-9 (PMC6934800; doi:10.1038/s41598-019-56408-9)
Supplement: Supplementary file 1 — Supplementary Video Figure Captions [file 41598_2019_56408_MOESM1_ESM.pdf]

Supplementary Information for:

ezTrack: An open-source video analysis pipeline for the investigation of animal behavior.

Pennington ZT, Dong Z, Feng Y, Vetere L, Page-Harley L, Shuman S, and Cai DJ

Supplementary Videos:

Supplementary Video 1: Tutorial for Location Tracking Module

Video demonstrating step by step how to use iPython files to implement Location Tracking Module in Jupyter Notebook. Copyright (c) 2017, Project Jupyter Contributors. All rights reserved. Redistribution and use in source and binary forms, with or withoutmodification, are permitted provided that the following conditions are met. Redistributions of source code must retain the above copyright notice, this list of conditions and the following disclaimer. Redistributions in binary form must reproduce the above copyright notice, this list of conditions and the following disclaimer in the documentation and/or other materials provided with the distribution. Neither the name of the copyright holder nor the names of its contributors may be used to endorse or promote products derived from this software without specific prior written permission. This software is provided by the copyright holders or contributors “as is” and any express or implied warranties, including, but not limited to, the implied warranties of merchantability and fitness for a particular purpose are disclaimed. In no event shall the copyright holder or contributors be liable for any direct, indirect, incidental, special, exemplary, or consequential damages (including, but not limited to, procurement of substitute goods or services,; loss of use, data or profits; or business interruption) however causes and on any theory of liability, whether in contract, strict liability, or tort (including negligence or otherwise) arising in any way out of the use of this software, even if advised of the possibility of such damage.

Supplementary Video 2: Tutorial for Freeze Analysis Module

Video demonstrating step by step how to use iPython files to implement Freeze Analysis Module in Jupyter Notebook. Copyright (c) 2017, Project Jupyter Contributors. All rights reserved. Redistribution and use in source and binary forms, with or withoutmodification, are permitted provided that the following conditions are met. Redistributions of source code must retain the above copyright notice, this list of conditions and the following disclaimer. Redistributions in binary form must reproduce the above copyright notice, this list of conditions and the following disclaimer in the documentation and/or other materials provided with the distribution. Neither the name of the copyright holder nor the names of its contributors may be used to endorse or promote products derived from this software without specific prior written permission. This software is provided by the copyright holders or contributors “as is” and any express or implied warranties, including, but not limited to, the implied warranties of merchantability and fitness for a particular purpose are disclaimed. In no event shall the copyright holder or contributors be liable for any direct, indirect, incidental, special, exemplary, or consequential damages (including, but not limited to, procurement of substitute goods or services,; loss of use, data or profits; or business interruption) however causes and on any theory of liability, whether in contract, strict liability, or tort (including negligence or otherwise) arising in any way out of the use of this software, even if advised of the possibility of such damage.

Supplementary Video 3: Example of Tracking with Location Tracking Module

Output video from ezTrack showing the stable tracking of an animal. Tracking continues when a foreign object enters into the field of view, providing a visualization of ezTrack being robust to distractors.

Supplementary Video 4: Example of syncing ezTrack output with Miniscope recording
Output video from ezTrack showing an animal running on a linear track (top) wearing a Miniscope recording calcium activity in the CA1 subregion of hippocampus. Below traces of cell activity (left) and processed Miniscope video with each cell color-coded (right).

Supplementary Video 5: Example of Tracking with Freeze Analysis Module
Output video from ezTrack showing measurement of freezing. Top portion of video shows original video and whether the animal is judged by ezTrack to be freezing. Bottom portion of video shows thresholded frame by frame pixel intensity differences, which ezTrack uses to calculate motion.

<u>Supplementary Figures:</u>

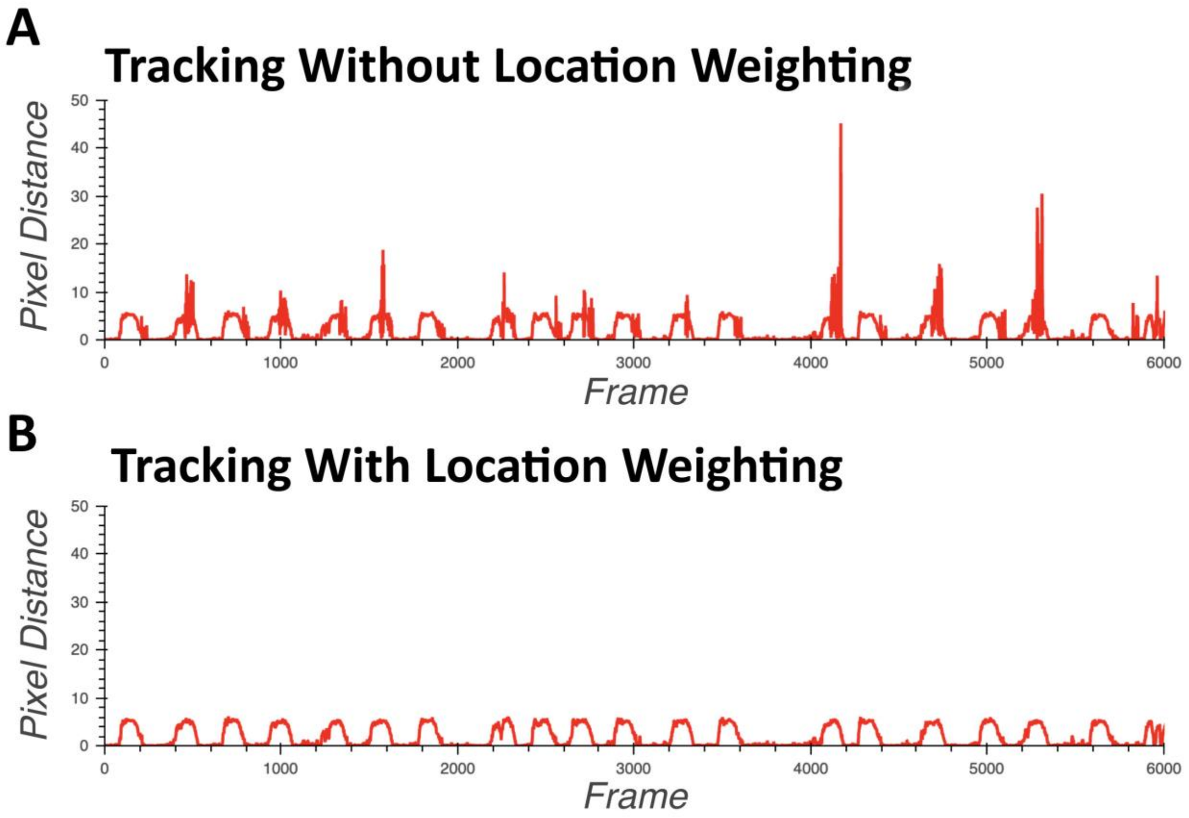

Supplementary Figure S1: Visualization of location tracking without and with location weighting.

Tracking of an animal running 20 trials on a linear track, either (A) without using ezTrack's window weighting procedure or (B) using the window weighting procedure. The experimenter's hand repeatedly moved into the field of view to pipette a liquid reward, and this resulted in several failures in tracking when the weighted window was not used, evidenced by large amplitude jumps in the distance travelled across the session.
